# Usefulness of ^18^F-FDOPA PET for the management of primary brain tumors: a systematic review of the literature

**DOI:** 10.1186/s40644-020-00348-5

**Published:** 2020-10-06

**Authors:** François Somme, Laura Bender, Izzie Jacques Namer, Georges Noël, Caroline Bund

**Affiliations:** 1 Nuclear medicine Department, Hautepierre University Hospital, 1, rue Molière, F-67000 Strasbourg, France; 2 Oncology Department, Hautepierre University Hospital, 1, rue Molière, F-67000 Strasbourg, France; 3 Strasbourg University, Unistra/CNRS UMR 7237, Strasbourg, France; 4 Radiotherapy Department, Paul Strauss Comprehensive Cancer Center, 3, rue de la porte de l’hôpital, F-67065 Strasbourg, France; 5 Strasbourg University, CNRS, IPHC UMR 7178, Centre Paul Strauss, UNICANCER, F-67000 Strasbourg, France

**Keywords:** F-DOPA, Glioma, Primary brain tumor, Systematic review

## Abstract

Contrast-enhanced magnetic resonance imaging is currently the standard of care in the management of primary brain tumors, although certain limitations remain. Metabolic imaging has proven useful for an increasing number of indications in oncology over the past few years, most particularly ^18^F-FDG PET/CT. In neuro-oncology, ^18^F-FDG was insufficient to clearly evaluate brain tumors. Amino-acid radiotracers such as ^18^F-FDOPA were then evaluated in the management of brain diseases, notably tumoral diseases. Even though European guidelines on the use of amino-acid PET in gliomas have been published, it is crucial that future studies standardize acquisition and interpretation parameters. The aim of this article was to systematically review the potential effect of this metabolic imaging technique in numerous steps of the disease: primary and recurrence diagnosis, grading, local and systemic treatment assessment, and prognosis. A total of 41 articles were included and analyzed in this review. It appears that ^18^F-FDOPA PET holds promise as an effective additional tool in the management of gliomas. More consistent prospective studies are still needed.

## Background

Management of primary brain tumors is based on contrast-enhanced magnetic resonance imaging (MRI). Despite progress in MRI as a perfusion and diffusion technique, it has a number of limitations (mainly due to the disruption of the blood–brain barrier), most particularly in differentiating recurrence from post-therapeutic effects [1, 2]. Additional magnetic resonance spectroscopy was developed to improve sensitivity and specificity. However, overlap between low-grade tumor values and those obtained for high-grade tumors has made this technique disputable [3, 4]. Its use to differentiate relapse and pseudo-progression remains under study [5].

Differentiating low-grade and high-grade features of a primary brain tumor as well as identifying patients with relapse and those with pseudo-progression are crucial in choosing the best treatment. Positron emission tomography with computed tomography (PET/CT) could be a tool to help reach these goals given the large number of radioisotopes that can be used in various clinical situations.

^18^Fluor-fluorodeoxyglucose (^18^F-FDG) PET/CT is widely used in oncology and can provide relevant information, even in the management of primary brain tumors [6, 7]. Nevertheless, a high rate of glucose metabolism in normal brain tissue (generating a poor signal-to-noise ratio with the tumor), tumors with low glucose metabolism (such as low-grade gliomas) and ^18^F-FDG’s lack of specificity remain limitations [8]. For these reasons, alternative PET radiotracers without these limitations have been evaluated quite recently in the management of brain tumors.

Amino-acid radiotracers have low uptake in normal brain tissue. Therefore, they more easily reveal the brain’s progressive processes, such as neoplastic disease. There are several radiolabeled amino acids currently in use. The first of them was ^11^C-methionine (MET); however, its use is limited because carbon 11’s half-life is 20 min and it is reserved for PET centers with an on-site cyclotron unit. Other amino-acid radiotracers labeled with fluor 18 have been synthetized, such as [18F]-fluoroethyl-tyrosine (^18^F-FET) and [18F]-L-dihydroxyphenylalanine (^18^F-FDOPA), which are easier to use in clinical routine because of their longer half-life (110 min). Studies have shown that ^18^F-FDOPA PET/CT is more accurate than ^18^F-FDG PET/CT and is more sensitive in detecting primary or recurrent gliomas [9–11]. Several studies have shown that these different amino-acid radiotracers (^18^F-FET, ^11^C-MET and ^18^F-FDOPA) performed equally well in the visual assessment of primary brain tumors [12–14].

Moreover, the cellular transport mechanism of amino-acid radiotracers is active, and their uptake seems to be correlated with the glioma grade. This is predominantly due to the increased transport of amino acids into tumor cells via an overexpression of the amino-acid transport system L (LAT 1 and LAT 2). Youland et al. showed a statistically significant positive correlation between ^18^F-FDOPA SUV_median_ and LAT1 expression (*p* = 0.04) [15].

Amino-acid radiotracers such as ^18^F-FDOPA have an impact on the management of patients with primary brain tumors [16]. Its use is now recommended by joint EANM, EANO and RANO guidelines in several indications [17]. However, it remains crucial that future studies standardize acquisition and interpretation parameters. This systematic review of the literature attempts to summarize the data on the usefulness of ^18^F-FDOPA PET for the diagnosis and management of primary brain tumors.

## Methods

A systematic review was conducted with reference to the PRISMA statement and the current methodological literature. Because of the heterogeneity of studies, performing a meta-analysis was not relevant.

The PubMed, ScienceDirect and Cochrane databases were searched for relevant English-language articles until July 2020. The search terms used were “DOPA” AND “PET” AND (“gliomas” OR “brain tumor”). The literature was systematically reviewed by two independent reviewers (LB and FS) using the Covidence tool.

## Results

### Study design

**Fig. 1:**
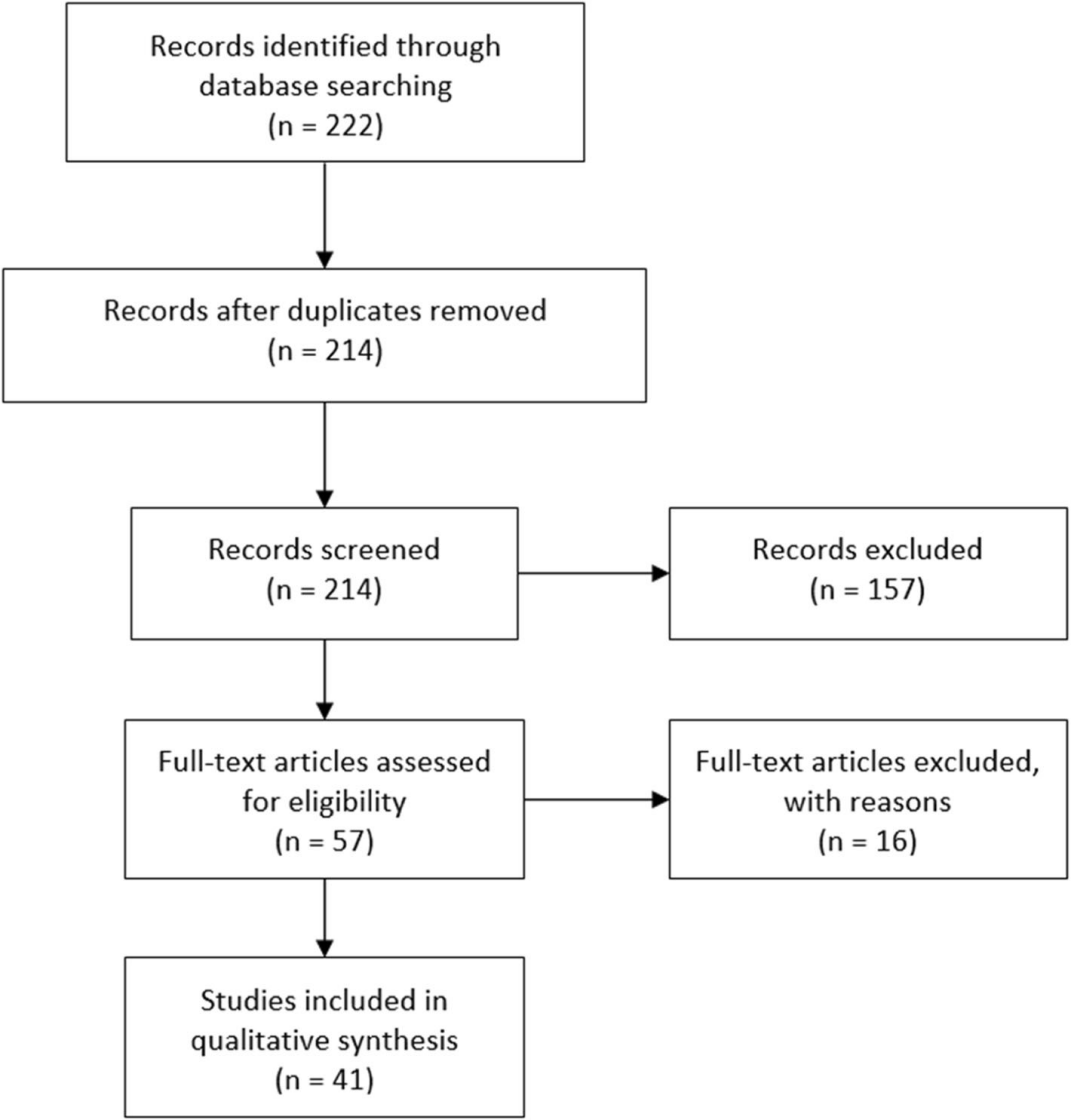
Flow chart of systematic review of the literature

A total of 222 articles were identified using the search strategy mentioned above. One hundred sixty-five articles were excluded for the following reasons: review articles, case reports, duplicates, irrelevant, abstract only, animal or pediatric studies, and articles in a language other than English. A total of 41 articles were finally included in the review (see Fig. 1).

**Fig. 2:**
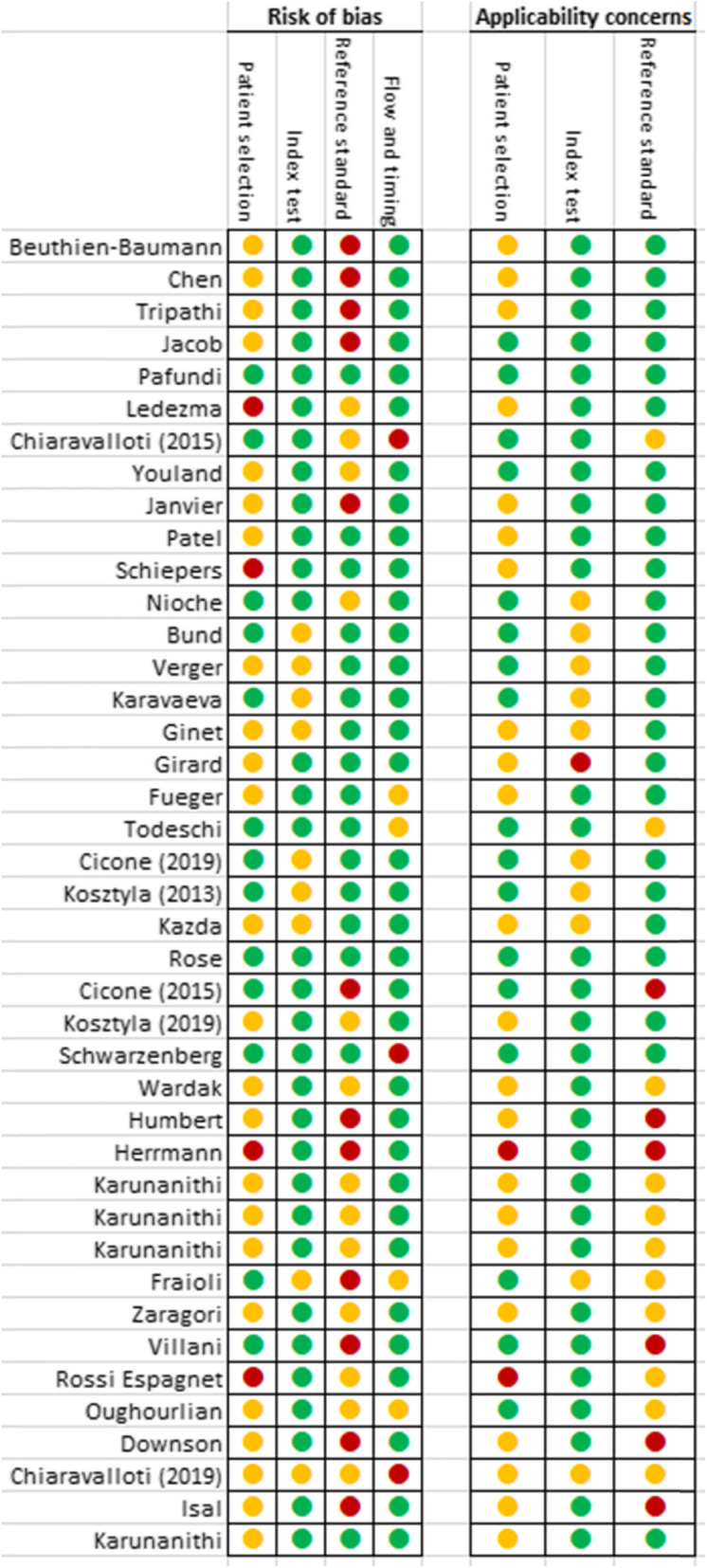
Summary of the risk of bias and applicability concerns according to the QUADAS-2 tool

**Fig. 3:**
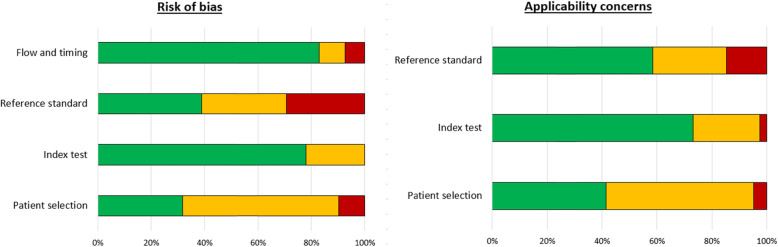
Graphic presentation of the risk of bias and applicability concerns according to the QUADAS-2 tool

The methodological quality of the studies included was evaluated using the Quality Assessment of Diagnostic Accuracy Studies tool (QUADAS-2) [18] by two independent reviewers (LB and FS). The results are summarized in Fig. 2 and Fig. 3. They show that in most studies (*n* = 28, 68%) patient selection can potentially introduce bias. This was mainly due to the small sample size of the population and the heterogeneity of the tumors included. Histological findings were almost always the reference standard defined in the studies included. However, only 13 studies (32%) were able to strictly correlate their results with histology. This could introduce bias in those articles as well.

### Disease detection

Several studies examined the sensitivity of ^18^F-FDOPA. Beuthien*-*Baumann et al. investigated 19 patients with suspected primary brain tumors. They obtained an overall sensitivity of 94% in correlation with histological or clinical and MRI follow-up [19]. The main limitation of this study was the lack of correlation with histological results: histological data were obtained for only eight patients (42%).

For the detection of primary brain tumor, other radioisotopes were compared with ^18^F-FDOPA. Chen et al. included 81 patients with newly diagnosed or recurrent brain tumors. All patients underwent both ^18^F-FDOPA and ^18^F-FDG imaging. Sensitivity, specificity, positive predictive value (PPV), negative predictive value (NPV) and overall accuracy were calculated. For ^18^F-FDOPA, the results were 98, 86, 95, 95 and 95%, respectively, and for ^18^F-FDG, they were 61, 43, 78, 25 and 57%, respectively [9]. Interestingly, with the criterion that any uptake above the background should be considered abnormal, using only visual inspection provided a higher sensitivity with ^18^F-FDOPA, but the specificity was as poor as that with ^18^F-FDG. Tripathi et al. prospectively included 15 patients suffering from low-grade gliomas. Among them, 13 patients (87%) with primary and recurrent low-grade gliomas had a visual uptake of ^18^F-FDOPA, whereas visual uptakes of ^18^F-FDG and ^18^F-FLT were observed in only 54 and 31%, respectively. Moreover, with a better tumor/normal brain uptake ratio (SUV_max_ T/N, defined by SUV_max_ of tumor divided by SUV_max_ of normal brain tissue), visual tumor delineation was easier with ^18^F-FDOPA [11]. However, due to the small number of patients included, the authors could not calculate sensitivity and specificity. Jacob et al. compared ^18^F-FDOPA, ^13^N-ammonia and ^18^F-FDG. The authors concluded that the sensitivity with ^18^F-FDOPA was substantially higher compared to the other radiotracers, but the figures and *p*-value were not provided. Furthermore, with only nine patients included, the number of cases was too small to draw statistical conclusions [10].

Some authors have demonstrated that ^18^F-FDOPA PET/CT can be compared favorably with contrast-enhanced MRI. Pafundi et al. published a prospective study including 10 patients and correlated 23 biopsy samples with ^18^F-FDOPA PET uptake and contrast enhancement on MRI images. The sensitivity and NPV of ^18^F-FDOPA PET and T1-CE MRI were 72.7% versus 27.3 and 14.3% versus 5.9%, respectively (*p*-value not available (NA)). Both modalities had 100% specificity and PPV. Overall accuracy was 73.9% for PET and 30.4% for T1 MRI (*p*-value NA) [20]. Ledezma et al. published a retrospective study, which included 91 patients and provided comparable results [21]. The authors split the patients into two groups: group 1 (*n* = 21), those patients who underwent ^18^F-FDOPA and MRI followed by tumor resection; group 2 (*n* = 70), those who lacked pathological confirmation. For group 1, the sensitivity of disease detection for PET was 95.2% compared to 90.5% for MRI. Interestingly, in one case, increased ^18^F-FDOPA activity was clearly detected in an area of nonenhancing tumor, a finding that may have been missed if MRI had been used alone. After a 7-month follow-up, imaging demonstrated significant tumor growth in this area (which also developed contrast enhancement) [21]. One problem reported in this study was the diagnosis of residual tumor using ^18^F-FDOPA after surgery. High levels of amino-acid transport into cells are also described for macrophages, which are activated after surgery. This might explain the mild ^18^F-FDOPA tracer uptake that can be present around resection cavities and should be interpreted with caution.

^18^F-FDOPA PET/CT could be helpful in the initial diagnosis of primary brain tumors, in addition to MRI. These two imaging modalities demonstrated that they could contribute complementary information. Moreover, in some cases where MRI remains uncertain (e.g., nonenhancing gliomas), the relatively new contribution of PET/MRI fused images could be a tool to obtain a noninvasive diagnosis [22]. Table 1 provides sensitivity, specificity, PPV, NPV and accuracy of ^18^F-FDOPA in the detection of primary brain tumors in each study included in this review.

**Table 1:** Performance of ^18^F-FDOPA in disease detection

Authors	Year	Patients (#)	Sensitivity (%)	Specificity (%)	PPV (%)	NPV (%)	Accuracy (%)	Optimal cut-off
Pafundi et al.	2013	10	72.7	100	100	14.3	73.9	NA (visual analysis)
Ledezma et al.	2009	21	95.2	NA	NA	NA	NA	NA (visual analysis)
Tripathi et al.	2009	15	100	NA	NA	NA	NA	NA (visual analysis)
Chen et al.	2006	81	98	86	95	95	95	T/S > 0.75
Beuthien-Baumann et al.	2003	19	94	NA	NA	NA	NA	NA (visual analysis)

*NA* Not available, *NPV* Negative predictive value, *PPV* Positive predictive value, *T/S* Tumoral uptake divided by striatum uptake

### Grading and correlation with histopathological features

In addition to tumor grade, the last World Health Organization (WHO) classification of gliomas dating from 2016 emphasizes integrating molecular parameters. In clinical practice, gliomas are split into two main groups with different prognoses: low-grade tumors (grade II) and higher-grade tumors (grades III and IV). Still, noninvasive categorization of tumor grade with image findings remains a challenge. ^18^F-FDOPA PET could be helpful in this matter.

Chiaravalloti et al. conducted a prospective study on 97 patients examining ^18^F-FDOPA uptake after surgery and radiotherapy. SUV_max_ and SUV_mean_ were significantly correlated with tumor grade (*p* < 0.05) [23]. As a limit, most of the patients underwent PET/CT a long time after surgery (mean, 41.48 months), when abnormal ^18^F-FDOPA uptake related to post-therapeutic inflammation had clearly decreased. Pafundi et al. published a prospective study with 10 patients. A significant correlation was found between SUV_max_ and tumor grade across biopsy samples (*p* = 0.0005). A significant difference was found between grade II and grade IV disease (*p* = 0.008) and between grade III and grade IV (*p* = 0.024) but not between grade II and grade III (*p* = 0.174) [20]. Similar results were also found in several other studies. Youland et al. correlated MRI and ^18^F-FDOPA PET/CT with tumor grade in 13 patients. Regions of MRI contrast enhancement correlated with the presence of high-grade recurrent tumor (*p* = 0.03), with 63% sensitivity and 80% specificity. A SUV_max_ T/N ratio greater than 2.0 (*p* = 0.0004) and SUV_max_ above 2.0 (*p* = 0.002) correlated with high-grade recurrent tumor with sensitivity at 85 and 80%, and specificity at 93 and 60%, respectively [24]. The inclusion of low- and high-grade gliomas in this series may influence the results, as the radiographic appearance of recurrent disease may be influenced by histology. Moreover, the low frequency of negative biopsies and the fact that the spatial distribution of MRI contrast enhancement is not clearly compared with ^18^F-FDOPA avidity can limit the robustness of this work. Janvier et al. found that all SUV-derived indices (SUV_max_, SUV_mean_, T/N ratios and tumor/striatum ratios (T/S, defined by tumoral uptake divided by striatum uptake)) were significantly correlated with tumor grade in 31 patients. The two best-correlated indices were SUV_mean_T/N (*p* = 0.001) and SUV_mean_T/S (*p* = 0.003). SUV_mean_T/N had a sensitivity of 71%, a specificity of 100%, and an area under curve (AUC) of 0.85 for an optimal threshold of 1.33 [25]. These results were significant considering only newly diagnosed gliomas since the authors failed to perform a significant subgroup analysis (newly diagnosed opposed to recurrent gliomas), mainly because they had only six recurrences in their study. Patel et al. retrospectively included 45 patients and correlated the uptake with numerous pathological data. They demonstrated that the SUV_max_ T/N was significantly higher in high-grade versus lower-grade glioma (*p* = 0.0002). The authors did not find a significant correlation between ^18^F-FDOPA uptake and the IDH mutation (*p* = 0.022) or the MGMT methylation (*p* = 0.66). However, the use of SUV_max_ could limit these results since it does not take into account the heterogeneity of the tumor [26].

Schiepers et al. evaluated static and dynamic ^18^F-FDOPA PET in 37 patients with brain tumors (33 primary brain tumors). Statistically significant differences (*p* < 0.01) for volume distribution of the radiotracer were found between newly diagnosed high-grade tumors and low-grade tumors and between newly diagnosed high-grade tumors and tumors with post-treatment changes [27]. The main limitation of this study was the heterogeneity of the tumors included (13 grade II, 10 grade III and 10 grade IV). The contribution of dynamic PET studies remains uncertain. A more recent study on 33 patients who underwent both static and dynamic ^18^F-FDOPA PET/CT showed that static PET/CT was able to determine the disease grade with 94% sensitivity and 66% specificity for a SUV_mean_ threshold of 2.5. Interestingly, all PET/CT indices were significant to distinguish between low-grade glioma and high-grade glioma in newly diagnosed tumors. Sensitivity and specificity were 90 and 70%, respectively. Considering recurrent tumors, only the SUV_mean_ index was significant (*p* < 0.001) to distinguish grade with 100% sensitivity and specificity. However, dynamic imaging did not significantly improve the diagnosis compared to static parameters [28].

Even when MRI evaluation is limited, notably in cases of nonenhancing tumors, ^18^F-FDOPA PET/CT contributed useful information. Bund et al. analyzed 33 patients with nonenhancing primary brain tumors. An optimal threshold of SUV_max_ T/*N* = 2.16 (AUC = 0.87) discriminated low-grade from high-grade gliomas with 60% sensitivity, 100% specificity, 100% PPV, and 83.3% NPV (*p* < 0.01) [29]. Moreover, the authors reported that ^18^F-FDOPA was also useful in the subgroup of low-grade gliomas. Indeed, it was able to discriminate between dysembryoplastic neuroepithelial tumor and grade II oligodendroglioma (*p* < 0.01).

A number of studies have evaluated the correlation of ^18^F-FDOPA PET with precise molecular features, such as mitotic activity (Ki-67 index) and the IDH mutation. Pafundi et al. found a significant correlation between SUV_mean_ and cellularity (*p* = 0.01) and an approaching significance with the Ki-67 index (*p* = 0.053). They used SUV_mean_ because it may be more representative of the entire cellular area used in calculations of both cellularity and Ki-67 than SUV_max_ [20]. Verger et al. included 43 patients with grades II and III gliomas before surgery. Surprisingly, patients with the IDH mutation showed higher ^18^F-FDOPA T/N (1.6 vs. 1.2; *p* = 0.046) and T/S ratios (0.9 vs. 0.6; *p* = 0.024) than patients without the IDH mutation. The authors discussed hypotheses to explain this unexpected result. Changes in metabolic pathways including increases in free amino acids, which lead to an increased activity of the amino-acid transporter and a better differentiation of gliomas were the main reasons suggested. Moreover, there was a significant positive correlation between ^18^F-FDOPA uptake and Ki-67 expression (*p* = 0.02) but not with presence/absence of 1p/19q co-deletion or ATRX loss of expression [30]. However, the retrospective design, the exclusion of glioblastoma and PET methodology (the authors used two-dimensional regions of interest to obtain the different ratios) were some of the limitations. In 29 patients with recurrent high-grade gliomas, Karavaeva et al. found a significant correlation between ^18^F-FDOPA PET SUV_mean_ and mitotic activity (*p* = 0.0362) [31]. It is important to note that biopsy samples were taken from MRI contrast-enhanced areas and were afterwards correlated with PET uptake. Another study reported similar results using additional dynamic PET acquisitions. Ginet et al. analyzed the correlation between the IDH mutation and the 1p/19q co-deletion with numerous static (SUV_max_, SUVmean, T/S, T/N and MTV) and dynamic (time to peak and slope) PET indices [32]. They found no correlation for static parameters but a significant correlation for both dynamic parameters. The best index was time to peak, both for the IDH mutation (*p* < 0.001, AUC = 0.789) and for 1p/19q co-deletion (*p* = 0.034, AUC = 0.679). Still, three main applicability concerns remain due to the use of carbidopa for some patients, the combination with MRI in cases of no radiotracer uptake (17% in this study) and a selection bias (patients were included only if both molecular data were known and a dynamic PET acquisition had been made). Moreover, it is worth noting that a recent study reported by Girard et al. sought to improve the time frame binning for dynamic ^18^F-FDOPA PET imaging [33]. The authors used a three-compartment model, which is the most commonly used for full kinetic analysis of PET, and compared five different time samplings in 14 patients. They reported that the average K1 value obtained by the 8 × 15 s–2 × 30 s–2 × 60 s–3 × 300 s time sampling was the closest to the target K1 value. Yet, variations in methodological factors such as ^18^F-FDOPA dose, a non-TOF PET system, image reconstruction or post-filtering could bias K1 estimates.

However, four studies highlighted various discrepancies. Considering the radiotracer’s uptake and the grade, three authors reported no correlation between those two parameters. Fueger et al. found a significant correlation between only newly diagnosed tumors and not in recurrent ones. Fifty-nine patients were analyzed. There was a significant correlation between uptake and grade and between uptake and the proliferation rate (Ki-67 index) only for newly diagnosed tumors. Uptake was significantly higher in high-grade than in low-grade tumors for newly diagnosed tumors (*p* = 0.005) but not for recurrent tumors (*p* = 0.22). Similarly, uptake correlated significantly with the Ki-67 index in newly diagnosed tumors (*p* = 0.001) but not in recurrent ones (*p* = 0.41) [34]. The main explanation reported by the authors was the wide range of blood–brain barrier breakdown indicators, depending on previous treatments, when considering recurrent tumors. This could be why correlation in these cases is relatively more uncertain. Chen et al. did not find a significant difference between uptake levels in 48 high-grade tumors and 18 low-grade tumors (*p* = 0.40) in a study of their 81 patients [9]. In a study evaluating the value of ^18^F-FDOPA PET in cases of nonenhancing MRI primary brain tumors, Todeschi et al. prospectively included 20 patients [35]. They reported an average SUV_max_ of 2.18 for high-grade and 2.025 for low-grade tumors, with no significant difference (*p* = 0.64).

Regarding the correlation between uptake and molecular data, Cicone et al. pointed out a number of contradictions. The authors studied 33 patients with gliomas. They did not find significant correlations between PET uptake parameters and the IDH mutational or the 1p/19 co-deletion status, neither for SUV_max_ (*p* = 0.56 and *p* = 0.29 respectively) nor SUVmean T/N (*p* = 0.32 and *p* = 0.82, respectively). The main limitation of this study was that almost all of the PET/CT examinations (94%) were made after the surgical procedure. This could have removed a portion of disease with uptake characteristics different from those of the remaining disease [36]. Zaragori et al. discussed this controversial result. The authors conducted a new analysis based on another cohort of 58 gliomas. The metabolic tumor volume (MTV) and T/N failed to differentiate IDH-wildtype from IDH-mutant (*p* > 0.5 for both) when considering the whole population. Nevertheless, when considering only diffuse gliomas (with the exclusion of glioblastomas), MTV was higher in IDH-mutant gliomas (*p* = 0.002) and a trend was observed for the T/N ratio (*p* = 0.1) [37]. Cicone et al. replied that MTV could not be considered as an uptake parameter and highlighted the trend found for T/N in the previous study [38].

Considering only newly diagnosed primary brain tumors, prior to any treatment, the data suggest that ^18^F-FDOPA PET is able to discriminate between low- and high-grade gliomas. Table 2 presents ^18^F-FDOPA PET sensitivity and specificity data to discriminate between low-grade gliomas and high-grade gliomas in each study included in this review.

**Table 2:** Optimal indices and cut-off to discriminate between low- and high-grade gliomas

Authors	Year	Patients (#)	Sensitivity (%)	Specificity (%)	Optimal ratio used
Patel et al.	2018	45	70	78	SUV_max_ T/*N* > 1.7
Youland et al.	2018	13	85	93	SUV_max_ T/*N* > 2
Bund et al.	2017	33	60	100	SUV_max_ T/*N* > 2.16
Janvier et al.	2015	31	71	100	SUVmean T/*N* > 1.33
Nioche et al.	2013	33	94	66	SUVmean > 2.5

*T/N* Tumor uptake divided by normal brain uptake.

### Target volume delineation and radiation treatment monitoring

Several studies showed that ^18^F-FDOPA PET could be a useful examination in the diagnosis of primary tumor (paragraph 3.2). The objective of using ^18^F-FDOPA PET/CT was then consistent with trying to include it in the management of local treatment (biopsy planning, radiotherapy).

Despite the lack of recommendations defining the biological target volume [17], several studies compared the delineation of target volumes obtained with MRI and ^18^F-FDOPA PET/CT. Pafundi et al. prospectively included 10 patients and correlated volume definition with histological findings. Each patient underwent one to three biopsies in concordant and discordant areas, uptaking ^18^F-FDOPA and contrast-enhancing MRI. For the six patients with T1-contrast enhancement, the percentage of ^18^F-FDOPA PET volume with a T/*N* > 2.0 outside the MRI contoured volume was on average 47.3% (range, 15.1–81.0%). The T2/FLAIR volume outside the high-grade threshold ^18^F-FDOPA PET uptake volume was on average 87.3% (range, 70.6–99.9%). These results suggest that there could be an impact of delivering a higher radiation dose into the volume delineated with ^18^F-FDOPA PET [20].

Other retrospective studies highlighted the low correlation between target volumes defined with MRI and PET/CT. Kosztyla et al. analyzed the interobserver variability and volume localizations, especially in cases of recurrent gliomas. Five observers contoured gross tumor volumes (GTVs) using MRI and PET/CT, and interobserver variability were quantified by the percentage of volume overlap. The mean interobserver volume overlaps for PET GTVs and MRI GTVs were not significantly different, 42% versus 41%, respectively (*p* = 0.67). The mean consensus volume was significantly larger for PET GTVs (58.6 cm^3^) than for MRI GTVs (30.8 cm^3^) (*p* = 0.003). Moreover, the percentage of the recurrence volume that extended beyond the PET GTV (52%) was significantly less than the percentage that extended beyond the MRI GTV (62%), (*p* = 0.04) [39]. Kazda et al. retrospectively included eight patients. The aim was to compare treatment planning with and without the incorporation of ^18^F-FDOPA PET imaging. For patients with contrast enhancement on T1-MRI (*n* = 5), biological target volumes (BTV_60Gy_) were less than 4.4 times as large as GTV_60Gy_; the planning target volume (PTV_60Gy_) including MRI + PET ranged from being the same to 1.8 times larger than PTV_60Gy_ using MRI only. For non-contrast-enhanced patients (*n* = 3), BTV_60Gy_ ranged from 48 to 202 times smaller than the GTV_60Gy_ (composed of the FLAIR MRI volume), while the resulting PTV_60Gy_ ranged from 3.2 to 72 times smaller. Interestingly, after inclusion of ^18^F-FDOPA PET biologic imaging, the average 60-Gy isodose volumes for the five patients with contrast enhancement increased 1.3-fold and decreased 2.5-fold in the three patients without contrast enhancement. All priority dose volume constraints for PTV_60Gy_ (V_100%_ ≥ 95% and V_110%_ < 0.5 cc) were met in both treatment plans for all patients, and all plans met critical organs at risk constraints (according to the Radiation Therapy Oncology Group) [40]. In the two preceding studies listed, the authors reported problems with the PET volume delineation. The physiological uptake of ^18^F-FDOPA in the basal ganglia may well interfere in the clear delineation of gliomas located near these structures. Moreover, postsurgical changes around the resection cavity can also exhibit radiotracer uptake and may have modified volume delineation. This may have added uncertainty to the study contours.

Certain authors also compared the target volume definition of ^18^F-FDOPA PET/CT with perfusion-weighted or diffusion-weighted MRI, with poor results in terms of volume overlap. Rose et al. prospectively analyzed 15 patients with newly diagnosed, confirmed high-grade gliomas. Two volumes were defined: regions of maximum ^18^F-FDOPA uptake within the tumor volume (voxels with the 20% highest SUV T/N ratio) and regions of minimum apparent diffusion coefficient (ADC) within the ^18^F-FDOPA-defined tumor volume. PET/CT volumes were significantly larger than ADC volumes (*p* = 0.0009). More importantly, considering the overlap between these two volumes, most patients presented with no or only modest overlap [41]. The authors suggested that regions of minimum ADC may primarily be associated with tumor ischemia, but there was no correlation with histological findings. This proposal needs to be evaluated with specific ischemia radiotracers (such as ^18^F-fluoromisonidazole). Approximately the same correlation was found with perfusion-weighted MRI. Cicone et al. defined tumor volume semiautomatically on ^18^F-FDOPA PET (threshold value, 1.6 over background) and was compared with the relative cerebral blood volume (rCBV) defined by perfusion-weighted MRI in 44 patients. ^18^F-FDOPA volume greatly exceeded rCBV volume (*p* < 0.00001). A median overlapping volume of 0.28 mL resulted in a 1.38% overall median spatial congruence. Interestingly, high-grade gliomas had a significantly larger ^18^F-FDOPA volume than low-grade gliomas (*p* = 0.023), which was not significant with rCBV volume (*p* = 0.071) [42]. No targeted biopsies were undertaken to confirm the results presented. Moreover, one might argue that the objective of perfusion-weighted MRI is not to define the precise tumor extent but instead to identify subregions of high-grade disease.

A recent paper evaluated the feasibility of dose-painted radiation therapy using ^18^F-FDOPA PET/CT. The authors included 10 patients with a high-grade glioma and analyzed the irradiation of the PTV with dose-painting (using MTV delineated with different thresholds). They demonstrated that the median volume of PTV receiving at least 95% of the prescribed dose was 99.6% with and 99.5% without dose painting (*p* = 0.5). There was no significant difference when considering the organs at risks as well. As limitations, this study included a small number of patients and the authors only used cell density to calculate the dose; they did not include the partial volume effect or the tumor hypoxia [43].

Several studies highlighted great differences between volumes defined with MRI and with ^18^F-FDOPA PET. Yet, study has demonstrated better outcome using amino-acid PET volume delineation. One prospective, multicenter, randomized phase II trial is currently in progress in an attempt to give an objective answer to this matter (NOA 10/ARO 2013–1) [44]. It is designed to test whether radiotherapy target volume delineation based on FET-PET improves progression-free survival (PFS) in patients with recurrent glioblastoma treated with re-irradiation, compared to target volume delineation based on MRI (NCT01579253).

### Chemotherapy and targeted therapy assessment

Considering systemic treatment monitoring in high-grade gliomas, there is currently a lack of data due to the limited effectiveness of using chemotherapy or targeted drugs in primary brain tumors. Multiple factors explain this: the impermeability of the blood–brain barrier is one of the most frequently suggested. However, there have been promising studies, especially in the evaluation of bevacizumab therapy. Bevacizumab is a recombinant humanized monoclonal antibody that blocks angiogenesis by inhibiting vascular endothelial growth factor A (VEGF-A). Schwarzenberg et al. showed that ^18^F-FDOPA PET/CT could identify early responders after 2 weeks of treatment with bevacizumab. Thirty patients were prospectively included. In multivariate analysis, the ^18^F-FDOPA MTV (defined by all voxels that fall within an SUV threshold determined by the mean SUV of the contralateral striatum) at 2 weeks (*p* < 0.05) and MTV changes at 6 weeks (*p* < 0.05) were the most significant predictors of overall survival (OS). ^18^F-FDOPA MTV change at 2 weeks (*p* < 0.01) was also the most significant predictor of progression-free survival [45]. However, the patients included had varying numbers of recurrences (median, 1.77). Since the number of recurrences is also predictive of survival, this could interfere with the results. Wardak et al. also identified a significant correlation between ^18^F-FDOPA PET/CT and overall survival. In their study, information from kinetic parameters (either from ^18^F-FLT alone, ^18^F-FDOPA alone, or both together; best adjusted R^2^ = 0.83) showed better predictive results than standardized uptake values (best adjusted R^2^ = 0.25) [46]. Consequently, the authors highlighted the need for dynamic PET/CT studies. This requirement leads to modifications in the acquisition procedures: starting the image acquisition simultaneously with radiotracer injection and a slightly longer acquisition time (about 35 min for dynamic images versus 20 min for the static acquisition).

PET indices such as metabolic tumor volume could be factors that predict early responders to systemic drugs, but larger prospective studies are required to confirm this assumption.

### Diagnosis between recurrence and post-therapeutic changes

The ability to distinguish between progression and post-treatment changes (mostly pseudo-progression within the first 12 weeks after completion of chemoradiotherapy or radionecrosis) is a major issue in the management of primary brain tumor. Indeed, it is recognized that MRI has limitations in diagnosing early recurrence [47]. However, RANO (response assessment in neuro-oncology) criteria are always based on MRI [48], even though issues remain [49]. ^18^F-FDOPA PET may help differentiate progression from post-treatment changes.

Many studies have attempted to evaluate the potential value of ^18^F-DOPA PET in this matter. A recent article by Humbert et al. evaluated the impact of PET/CT through a multidisciplinary brain tumor board. A first decision was made with clinical and MRI data, without knowing the results of ^18^F-DOPA PET/CT; then a second decision was made with the inclusion of PET/CT. The authors demonstrated that this technique was able to modify the diagnosis or the therapeutic strategy in up to 33.3% of cases when considering patients with glioblastomas. The main limitation was the lack of correlation with pathological data (9.4% of patients) [50]. Youland et al. correlated 37 stereotactic biopsies and histological results from 13 patients according to areas of increased ^18^F-FDOPA uptake and areas of MRI contrast enhancement. To distinguish between radionecrosis and recurrence, MRI sensitivity and specificity were 52 and 50%, respectively, ^18^F-FDOPA PET sensitivity and specificity were 82 and 50%, respectively [24]. Herrmann et al. analyzed 110 patients retrospectively. Images were correlated with histological data in 41 (37.3%) cases and clinical and imaging follow-up in 69 (62.7%) cases. The authors did not separate the two groups in their results. Overall, visual analysis resulted in sensitivity, specificity, accuracy, and positive and negative predictive values of 85.2, 72.4, 81.8, 89.6 and 63.4%, respectively, considering ^18^F-FDOPA PET/CT. All PET indices (SUV_max_, SUV_mean_, T/N ratios and T/S ratios) were significantly higher in progressive than in nonprogressive patients. Interestingly, semiquantitative image analysis did not improve accuracy over visual PET/CT image analysis, with the AUC ranging from 0.77 to 0.82 versus 0.82 for visual analysis [51]. Nevertheless, several limitations remain in this study: the retrospective design, the fact that different PET systems were used, which might have affected SUV measurements (even if checks were made with phantoms) and a potential selection bias since patients were included based on a positive MRI diagnosis of recurrent disease. Karunanithi et al. prospectively included 35 patients comparing ^18^F-FDOPA PET/CT and MRI to detect recurrence. Sensitivity, specificity and overall accuracy were, for ^18^F-FDOPA PET/CT, 100, 88.9 and 91.1%, respectively, compared to, for contrast-enhanced MRI, 92.3, 44.4 and 80%, respectively [52].

^18^F-FDOPA PET/CT was also compared with other radiotracers developed for SPECT/CT and PET/CT. The most frequently used was obviously ^18^F-FDG. Karunanithi et al. prospectively compared 28 patients who underwent PET/CT with both ^18^F-FDOPA and ^18^F-FDG. The sensitivity, specificity and accuracy of ^18^F-FDG PET/CT were 47.6, 100 and 60.7%, respectively, and those for ^18^F-FDOPA PET/CT were 100, 85.7 and 96.4%, respectively. The difference in the findings between ^18^F-FDG PET/CT and ^18^F-FDOPA PET/CT was significant (*p* = 0.0005) [53]. ^18^F-FDOPA also showed better performance than radiotracers developed for SPECT/CT, such as ^99m^TC-GH. Karunanithi et al. prospectively compared these two tracers in 30 patients. Sensitivity, specificity, and accuracy were 86.4, 62.5 and 80% for ^99m^Tc-GH SPECT/CT and 100, 87.5 and 96% for ^18^F-FDOPA PET/CT, respectively [54]. All results reported by Karunanithi et al. in their three studies may present certain limitations: the sample size was relatively small, especially considering low-grade gliomas, and a confirmation of recurrence by histological findings was obtained for a small number of cases.

More recently, Fraioli et al. took an interest in the innovative PET/MRI technique [55]. They prospectively compared 40 residual tumor volumes (obtained from PET and MRI) and several PET (SUV_max_, T/N and T/S) and MRI (relative cerebral blood volume and relative cerebral blood flow) parameters. PET volumes were significantly larger than those obtained from MRI, both for low-grade and high-grade tumors (*p* = 0.02 and *p* = 0.0002, respectively). Both modalities were concordant in 37 patients (93%). A single-modality analysis of PET imaging demonstrated an AUC of 0.94. A combined multiparameter PET/MRI approach resulted in an AUC of 0.99, showing that MRI and F-DOPA are complementary modalities for assessment of tumor burden. However, there was no pathological confirmation of residual tumor and a dichotomous evaluation of presence or absence of active disease was made (without taking into consideration other important relevant information).

Lastly, Zaragori et al. assessed dynamic acquisition in this issue by evaluating the predictive value of static (SUV_max_, SUVmean, T/S and T/N) and dynamic parameters (time to peak and slope) in terms of recurrence and survival. Except time to peak, all the PET parameters studied were significant univariate predictors of glioma recurrence (*p* < 0.001) and the T/S ratio was the sole significant parameter in the multivariate analysis. No indices were predictive of overall survival, whereas all PET parameters, except time to peak, were correlated with progression-free survival. Thus, the authors showed that none of the dynamic parameters provided any additional diagnostic information. As limitations, Zaragori et al. were able to obtain a pathological confirmation of recurrence for only four patients and the authors mixed low- and high-grade gliomas that may have benefited from different therapeutic strategies, which could have influenced survival [56].

^18^F-FDOPA PET should be considered as a complementary tool to assess real progression when MRI remains uncertain. Table 3 collects the sensitivity, specificity and accuracy of ^18^F-FDOPA PET in the diagnosis of recurrence and post-therapeutic changes in each study included in this review.

**Table 3:** Performance of ^18^F-FDOPA in discriminating recurrence from post-therapeutic effects

Authors	Year	Patients (#)	Sensitivity (%)	Specificity (%)	PPV (%)	NPV (%)	Accuracy (%)	Cut-off
Zaragori et al.	2020	51	97.1	94.1	NA	NA	96	T/S > 1
Youland et al.	2018	13	82	50	NA	NA	NA	T/N > 2.0
Karunanithi et al.^a^	2014	30	100	87.5	NA	NA	96	T/S > 0.6
Karunanithi et al.^a^	2013	35	100	88.9	NA	NA	97.1	NA (visual)
Herrmann et al.	2013	110	85.2	72.4	89.6	63.4	81.8	T/*N* > 1.81
Karunanithi et al.^a^	2013	28	100	85.7	NA	NA	96.4	T/N > 1.3

*NA* Not available, *NPV* Negative predictive value, *PPV* Positive predictive value, *T/N* Tumor uptake divided by normal brain uptake, *T/S* Tumor uptake divided by striatum uptake

^a^: the results of these studies are based on the same patient population

### Prognostic value

The ability to sort patients into subgroups of different prognoses is important. It can help the clinician adapt or even change treatment. In this indication, ^18^F-FDOPA PET could have a place to claim. Many authors have examined this issue, resulting in several PET indices positively correlated with survival. In this section, we will summarize the most significant indices objectified in studies.

Villani et al. prospectively included 50 grade II gliomas. After a median follow-up of 16 months, on multivariate analysis, a maximum standardized uptake value greater than 1.75 (*p* = 0.005) was an independent predictor of disease progression [57]. Correlation with overall survival was not calculated because patient follow-up was too short. On multivariate analysis, another study considering 12 patients with low-grade gliomas demonstrated a significant correlation between follow-up status (stable versus disease progression at 1 year) and T/N with a cut-off > 1.7 (*p* = 0.05 [58];. In addition to the small population examined in this study, there was a majority of oligodendroglioma cases (eight patients, 67%). As already shown in the literature, this histological subtype may have a specific presentation pattern. Indeed, it may show increased amino-acid uptake and high rCBV values that are not related to tumor grade but more consistently related to 1p/19q co-deletion [59]. Another retrospective study was conducted in 27 low-grade gliomas [60]. The authors analyzed the rates of change in FLAIR volume and in ^18^F-FDOPA SUV_max_ normalized to the basal ganglia (nSUV_max_). General linear models were used to integrate clinical information (age and treatment) with MRI and PET measurements to predict malignant transformation. A model using age, treatment, rate of change in FLAIR volume and in radiotracer uptake predicted a malignant transformation within 6 months (*p* = 0.0248). Moreover, only the rate of change in radiotracer uptake was correlated with overall survival in the multivariate analysis (*p* = 0.0033). The limited number of patients, the retrospective nature of the study and the fact that PET and MRI scans were not made at a specific controlled interval could limit the applicability of this study.

The prognostic impact of ^18^F-FDOPA PET was also highlighted when considering all grades of gliomas. Patel et al. demonstrated that age (*p* = 0.001) and the metabolic tumor volume on PET (*p* = 0.016, using a SUV_max_ T/N threshold) were correlated with the 2-year overall survival time, in multivariate analysis [26]. Dowson et al. studied radiotracer uptake in nine patients, at baseline, immediately before tumor resection and 12 weeks after resection. The results demonstrated that a decrease in ^18^F-FDOPA uptake (ΔSUV_max_) is a predictor of extended survival (*p* = 0.002) [61]. The population size was very small in this study and could limit its statistical power. Nevertheless, a recent study reported results that are more restrained. Chiaravalloti et al. retrospectively included 133 primary brain tumors [62]. They correlated the OS and the PFS with the SUV_max_ and the SUVr (defined by the SUV_max_ of the tumor divided by the SUV_max_ of the contralateral occipital region). In the whole cohort, the uptake was significantly correlated with OS (*p* = 0.01) but not with PFS. These two indices were correlated with OS and PFS (*p* = 0.03 and *p* = 0.007, respectively) for grade II gliomas and no correlations were found for grade III and grade IV gliomas. Several limitations remain: a high proportion of glioma without radiotracer uptake (*n* = 41) and a large interval between the surgical intervention and the PET/CT (low-grade glioma could have switched to a more malignant grade).

Several authors tried to correlate ^18^F-FDOPA PET/CT with MRI findings and survival. Isal et al. compared T/N with velocity of diameter expansion (VDE), calculated on MRI, a known prognostic factor [63]. A ratio higher than 1.8 was significantly more frequent in patients with a VDE < 4 mm compared to those with a VDE ≥ 4 mm (45% vs 0%, *p* = 0.04) [64]. The tumor growth rate was chosen as a surrogate for clinical course in this study, since the overall survival could not be obtained owing to the long clinical course of low-grade glioma.

When considering only recurrent gliomas, Karunanithi et al. prospectively included 33 patients. After a median follow-up of 20.2 months, on multivariate analysis, only size of the recurrent tumor on MRI (*p* = 0.002) and the T/N ratio of ^18^F-FDOPA PET (*p* = 0.005) were found to be independent predictors of survival [65]. Another large retrospective study analyzed 110 patients with a median follow-up of 34.9 months. All PET indices were significant predictors of progression-free survival, with the mean lesion-to-T/N ratio providing the best discrimination (*p* < 0.001). Conversely, none of the parameters investigated were predictive of overall survival [51].

When examining all the studies included in this review, it appears that ^18^F-FDOPA PET can help stratify patients into subgroups of different prognoses. Several PET indices were exploited; the most frequently used is SUV_max_ T/N. However, there is still a huge diversity of PET indices used, which limits comparison between studies. Nevertheless, these results need to be confirmed by larger prospective studies. We have summarized the best indices and cut-offs found in each study in Table 4.

**Table 4:** Optimal indices and cut-off that correlated with prognosis

Authors	Year	Patients (#)	Population	Optimal index and cut-off	***P***-value
Chiaravalloti et al.	2019	133	II = 68, III = 34, IV = 31	SUVr > 1.37	0.01
Isal et al.	2018	20	II = 13, III = 7	SUV_max_ T/*N* > 1.8	0.04
Rossi Espagnet et al.	2016	12	II = 12	SUV_max_ T/N > 1.7	0.05
Villani et al.	2015	50	II = 50	SUV_max_ > 1.75	0.005
Dowson et al.	2014	9	IV = 9	ΔSUV_max_ > 4.74	0.002
Herrmann et al.	2014	110	III = 33, IV = 77	SUVmean T/S > 1.06	< 0.001
Karunanithi et al.	2014	33	I = 2, II = 9, III = 6, IV = 16	SUV_max_ T/*N* > 1.51	0.005

*SUVr* Tumor uptake divided by contralateral occipital uptake, *T/N* Tumor uptake divided by normal brain uptake, *T/S* Tumor uptake divided by striatum uptake.

## Conclusion

Due to the poor prognosis of gliomas, especially considering high-grade tumors, their management remains a huge challenge. It is widely accepted that ^18^F-FDOPA PET can provide useful information in terms of initial diagnosis and the extent of gliomas in the context of recurrent tumors. This systematic review gathers the current data reported in the literature regarding the use of ^18^F-FDOPA in the other steps of primary brain tumor management. Nevertheless, standardization of acquisition and interpretation parameters (as addressed by EANM, SNNMI and EANO) remains essential.

## Data Availability

Not applicable.

## Abbreviations

MRI: Magnetic resonance imaging
PET/CT: Positron emission tomography with computed tomography
SUV: Standardized uptake value
WHO: World Health Organization
AUC: Area under the curve
GTV: Gross tumor volume
PTV: Planning target volume
ADC: Apparent diffusion coefficient
rCBV: Relative cerebral blood volume
VEGF-A: Vascular endothelial growth factor A
MTV: Metabolic tumor volume
RANO: Response assessment in neuro-oncology
VDE: Velocity of diameter expansion
